# Prediction of the severity of obstructive sleep apnea by anthropometric features via support vector machine

**DOI:** 10.1371/journal.pone.0176991

**Published:** 2017-05-04

**Authors:** Wen-Te Liu, Hau-tieng Wu, Jer-Nan Juang, Adam Wisniewski, Hsin-Chien Lee, Dean Wu, Yu-Lun Lo

**Affiliations:** 1 Division of Pulmonary Medicine, Department of Internal Medicine, Shuang Ho Hospital, Taipei Medical University, New Taipei City, Taiwan; 2 Department of Internal Medicine, School of Medicine, College of Medicine, Taipei Medical University, Taipei, Taiwan; 3 School of Respiratory Therapy, College of Medicine, Taipei Medical University, Taipei, Taiwan; 4 Department of Engineering Science, National Cheng Kung University, Tainan, Taiwan; 5 Sleep Science Center, Taipei Medical University Hospital, Taipei Medical University, Taipei, Taiwan; 6 Sleep Center, Shuang Ho Hospital, Taipei Medical University, New Taipei City, Taiwan; 7 Department of Mathematics, University of Toronto, Toronto, ON, Canada; 8 Department of Psychiatry, Shuang Ho Hospital, Taipei Medical University, New Taipei City, Taiwan; 9 Department of Psychiatry and Medical Humanities, School of Medicine, College of Medicine, Taipei Medical University, Taipei, Taiwan; 10 Department of Neurology, Shuang Ho Hospital, Taipei Medical University, New Taipei City, Taiwan; 11 Department of Neurology, School of Medicine, College of Medicine, Taipei Medical University, Taipei, Taiwan; 12 Department of Thoracic Medicine, Healthcare Center, Chang Gung Memorial Hospital, School of Medicine, Chang Gung University, Taoyuan, Taiwan; Tianjin University, CHINA

## Abstract

To develop an applicable prediction for obstructive sleep apnea (OSA) is still a challenge in clinical practice. We apply a modern machine learning method, the support vector machine to establish a predicting model for the severity of OSA. The support vector machine was applied to build up a prediction model based on three anthropometric features (neck circumference, waist circumference, and body mass index) and age on the first database. The established model was then valided independently on the second database. The anthropometric features and age were combined to generate powerful predictors for OSA. Following the common practice, we predict if a subject has the apnea-hypopnea index greater then 15 or not as well as 30 or not. Dividing by genders and age, for the AHI threhosld 15 (respectively 30), the cross validation and testing accuracy for the prediction were 85.3% and 76.7% (respectively 83.7% and 75.5%) in young female, while the negative likelihood ratio for the AHI threhosld 15 (respectively 30) for the cross validation and testing were 0.2 and 0.32 (respectively 0.06 and 0.1) in young female. The more accurate results with lower negative likelihood ratio in the younger patients, especially the female subgroup, reflect the potential of the proposed model for the screening purpose and the importance of approaching by different genders and the effects of aging.

## Introduction

Obstructive sleep apnea (OSA) is a common disorder. The prevalence of OSA is approximately 14% for adult men and 5% for adult women.[1, 2] Patients with OSA suffer from the intermittent cessation or reduction of breathing during sleep due to upper airway collapse with sleep fragmentation and/or oxygen desaturation, which results in non-restorative sleep, excessive daytime sleepiness, and fatigue.[3] Also, more evidence shows that OSA is associated with several different diseases, ranging from cardiovascular diseases[4] to neurocognitive impairment[5] to stroke;[6] OSA has also been established as the cause for several public disasters.[7] Although OSA has received a great amount of attention in recent decades, many patients with OSA (approximately 80–90% of all patients) are under-diagnosed and even untreated.[1, 8]

The diagnosis of OSA is based on the relevant clinical features and the objective demonstration of sleep-disordered breathing. When OSA is suspected, in-laboratory polysomnography (PSG) is the first-line diagnostic study. The recorded multichannel signals provide the information regarding sleep status, respiratory disturbance, and gas exchange abnormalities.[9] While it is a common practice and provides the gold standard of diagnosis, it is nonetheless not feasible either practically or economically for screening purposes. Due to the requirement of a full sleep laboratory and a trained technician, it is labor-intensive and time-consuming. The portable device, or small polygraphy, is a common alternative to PSG for the OSA diagnosis. Although it is less labor-intensive, the patients should satisfy several conditions, for example, they should have high pretest probability of having moderate to severe OSA and do not have medical comorbidities or other sleep disorders [10]. Thus, a good screening tool is needed for using this kind of portable device. Given its various impacts on public health, finding a different and efficient way to screen patients for OSA is an important health issue.[11]

In the primary care setting, several surrogates have been proposed to achieve the screening purpose based on various clinical prediction rules and scores, which evaluate the common symptoms and signs of OSA. Such examples include the Berlin questionnaire (BQ), Sleep apnea clinical score (SACS), and the STOP-Bang questionnaire. As useful as these questionnaires are, however, these questionnaires are mainly designed around data from Caucasian subjects, which might not be directly applicable to other races.[12, 13] Since the populations are increasingly becoming more heterogeneous in this era of globalization, the utility of these questionnaires for screening may become increasingly limited. A different approach is thus needed.

It is known that anthropometric features have significant effects on the OSA severity. One usual measurement is body fat composition.[14, 15] Body shape profiles, like neck and waist circumference, neck-to-waist ratio, waist-to-height ratio, and waist-to-hip ratio, have been found to be associated with the severity of OSA.[16–18] Recently researchers have examined the anthropometrics of patients with OSA by assessing gender-related differences in adipose tissue distribution, and these studies have concluded that new models for gender-specific OSA phenotypes are needed.[19–23] As measurements of these anthropometric features are readily available and easy to obtain in the outpatient setting, it is interesting to ask if we can predict OSA severity based on anthropometrics. However, to the best of our knowledge, there has been no large-scale study examining the potential of screening OSA severity by combining different body shape profiles.

In sum, we need an applicable method to establish prediction models for different races based on easy-to-obtain parameters, and body shape profiles are suitable choices. Support vector machine (SVM) [24] is a supervised machine learning algorithm (nonlinear regression technique) that has the ability to extract complicated relationship between parameters, and its properties have been extensively studied. In this study, to answer the above questions, we establish a prediction model for Asians by taking body shape profiles and age into account via SVM. We hypothesize that the established predictors are accurate for OSA severity. To confirm this hypothesis, we collected two large patient databases from two independent sleep labs and designed a prediction model for the OSA severity based on body shape profiles.

## Methods

### Patient information

Patient information was collected from two independent sleep centers at the Taipei Medical University Hospital (TMUH, Taipei, Taiwan) and Shuang-Ho Hospital (SHH, New Taipei City, Taiwan). The inclusion criteria for this study were patients referred for suspected sleep disordered breathing, ages between 20 and 80, recordings available from at least one overnight PSG and a completed questionnaire battery including the informed consent. The exclusion criteria were PSG recordings for continuous positive airway pressure ventilation titration, patients were hospitalized and/or poor sleep efficiency (less than 40%). The joint institutional review board of the Taipei Medical University approved the study protocol (TMU-JIRB No.: 201412036). All sleep signals were acquired on the ResMed Embla N7000 or on the Sandman Elite data acquisition system. All sleep apnea and hypopnea events were scored by experienced sleep technologists according to the AASM 2007 guidelines. Apneas were defined as a drop in the peak thermal sensor excursion by ≥ 90% from baseline with a minimum duration of 10 seconds. Hypopneas were scored when the nasal pressure signal excursion dropped by ≥30% of baseline with a minimum duration of 10 seconds and associated with ≥ 4% oxygen desaturation from pre-event baseline. [25]

The patients in the TMUH database were enrolled from Oct. 2005 to Apr. 2014. The TMUH database contains the following parameters: Age, sex, weight and height, Epworth Sleepiness Scale (ESS), BQ as well as PSG examination data. Furthermore, the anthropometric features such as head, buttock, waist and neck circumferences were also measured in the sleep lab before performing the PSG. The patients in the SHH database were enrolled from Oct. 2013 to Sept. 2014. The SHH database contains the following parameters: Age, sex, weight and height, waist and neck circumferences, ESS, and PSG examination data. The BQ is composed of three categories including snoring behaviour, waketime sleepiness or fatigue, and obesity and/or hypertension. Each of these categories is classified as severe or not severe. If two out of three categories are severe, then the patient is of high risk with BQ number 1; otherwise the patient is of low risk with BQ number 0.[26] While the sleep experts in these two sleep labs followed the same protocol and standard to evaluate subjects, these two sleep labs are independent and different parameters were collected.

### Model build-up and statistics

We include parameters based on the clinical needs, so no parameter selection is needed. For each parameter, we report the Spearman’s correlation coefficient with the severity.

To classify the severity of sleep apnea from the body profile parameters, we applied the kernel support vector machine (SVM) [24] to capture the potential nonlinear structure hidden inside the predictors. The SVM model could be viewed as a nonlinear regression model, which has the following format:

$$c=\sum_{i=1}^{n}\alpha_{i}\;k(x_{i},x)+b,$$ (1)

where *c* is the output of the SVM of the new patient data *x*, which could be interpreted as the posterior “probability” or score of the classifier, *x*_*i*_,*i* = 1…*n*, is the training dataset, *α*_*i*_ and *b* are model parameters determined by the SVM algorithm [26]. We classify the severity of a new patient *x* based on the output value *c* and a pre-determined threshold value–if *c* is greater than the threshold, we assign *x* to group 1, otherwise group 2. We set the threshold value for *c* to be -2 to favour the sensitivity for the screening purpose in this study. To evaluate the SVM model, we chose the Gaussian radial basis function as the kernel function, *k*, with the scaling factor 0.5 and the box constraint for the soft margin set to 1. Each predictor was normalized by subtracting the median and dividing by the median absolute deviation before evaluating the SVM model.

To prevent over-fitting and to validate the classification results, we divided the statistical analysis into the training and validation step and the testing step.[24] In the training and validation step, we analyzed the TMUH database by repeating random sub-sampling validation 100 times. We randomly partitioned the data into the training dataset and the testing dataset, with the training dataset being created by randomly selecting 90% of the patients while the remainder serving as the testing dataset, and repeated it for 100 times. The average and standard deviation are reported, and the model is established. The unbalanced data is handled by the rescaling scheme—it is automatically rescaled by (N1+N2)/(2*N1) for the data points of group one and by (N1+N2)/(2*N2) for the data points of group two, where N1 is the number of elements in group one and N2 is the number of elements in group two. The trained classifier was then applied to predict the AHI of patients in the SHH dataset.

We report the binary classification results according to sensitivity, specificity, accuracy, and the area under curve (AUC). For the screening purpose, we also report the negative likelihood ratio and the pre-test probability (prevalence) of the sleep apnea. Results are expressed as a mean with standard deviation unless otherwise stated. Patient characteristics were analyzed using the univariate analysis or the chi-square test. The differences of two independent correlations were analyzed using Fisher’s z-transform. Differences in the prediction accuracy between any two chosen groups were analyzed with the Mann-Whitney U test. A value of p <0.05 was considered statistically significant. Multiple significant tests were calculated with Bonferroni correction. We applied the statistics toolbox of Matlab R 2014b to carry out SVM and other statistics.

## Results

In the TMUH database, we recruited 7386 patients and excluded 2141 patients: 361 were younger than 20 years or older than 80 years, 39 had poor sleep efficiency (less than 40%), 55 patients were hospitalized, 1103 had continuous positive airway pressure ventilation titration, and 583 had missing data. In the end, we had 5245 patients, among whom 4003 were males and 1242 females. We classify the severity of OSA by two thresholds, 15 and 30. For each threshold, the group greater and less than the threshold were respectively labeled as being positive (group 1) and negative (group 2). The distribution of demographic information and collected parameters are listed in Table 1.

**Table 1:** The distribution of demographic information and collected parameters in the TMUH and SHH databases. AHI: Apnea hypopnea index; BMI: Body mass index; BQ: Berlin questionnaire; ESS: Epworth sleepiness scale; *: the p-value is less than 0.05 between male and female within the TMUH database; #: the p-value is less than 0.05 between male and female within the SHH database.

	TMUH		SHH	
	Male	Female	Male	Female
Number (total)	4003	1242	816	338
Number (AHI<15)	1141	859	298	235
Number (30>AHI≥15)	730	205	158	57
Number (AHI≥30)	2132	178	360	46
AHI (events/hour)	37.2±28.1*	13.5±19.1*	32.3±27.5^#^	14.2±17^#^
Age (years)	45.3±12.8	45.4±14	46.6±12.9^#^	49.5±13.8^#^
BQ	0.2±0.4*	0.3±0.7*	-	-
ESS	10.1±5.8*	8.6±5.6*	10.4±5.5^#^	9.6±6.1^#^
Head (cm)	57±2.1*	54.7±2.1*	-	-
Buttock (cm)	102±8.9*	98.1±11.7*	-	-
Neck (cm)	39±3.4*	33.4±3.4*	38.7±3.5^#^	33.5±3.3^#^
Waist (cm)	94.1±12.3*	81.8±14.3*	95.4±12.3^#^	85.7±11.9^#^
BMI (kg/m^2^)	27.1±4.9*	25±6.3*	27.7±5^#^	25.8±5.1^#^

In the SHH database, we recruited 1572 patients, and excluded 418 patients among whom 61 were younger than 20 years or older than 80 years, 21 had poor sleep efficiency (less than 40%), 3 were hospitalized, 231 had continuous positive airway pressure ventilation titration, and 102 had missing data. In the end, we had 1154 patients (816 males and 338 females). The distribution of demographic information and collected parameters of the SHH database are listed in Table 1. For the male and female subgroups, with the exception of BMI, the parameters AHI, Age, Neck, and Waist were not statistically different from those in the TMUH database.

### Determining the body-profile model

For the body profile model, we considered two subgroups: males and females. In the TMUH database, the Spearman correlation coefficients of different parameters with the sleep apnea severity in different groups are listed in Table 2. The body profile parameters, age and ESS can be considered to correlate well with the outcome, while the correlations in the female and male groups were significantly different for the ESS, waist, age, and BMI parameters. For the convenience of clinical practice, we considered the three most correlated body profiles: waist circumference, neck circumference, and BMI. For the non-body profile parameters, age had a strong correlation in the female group. As a result, three body profiles parameters (waist circumference, neck circumference, and BMI) and age were included to build up the prediction model by the SVM. We call this model the *body profile model*.

**Table 2:** The Spearman correlation coefficients of different collected parameters with the sleep apnea severity in the TMUH database. Three subgroups are selected from the database–all patients, males only and females only. BQ: Berlin questionnaire; ESS: Epworth sleepiness scale; BMI: Body mass index; *: the p-value is less than 0.05 between male and female within the TMUH database.

	Age	BQ	ESS	Head	Buttock	Neck	Waist	BMI
All	0.23	-0.06	0.23	0.29	0.45	0.58	0.6	0.5
Male	0.2*	-0.02	0.23*	0.15	0.42	0.47	0.52*	0.47*
Female	0.4*	-0.03	0.13*	0.16	0.45	0.5	0.57*	0.52*

The cross validation results from the TMUH database and the testing results of the body profile model on the SHH are shown in Table 3. The prediction accuracy of the female group is better than that of the male group, particularly the AUC and LR+ in the testing group. This finding justifies the applicability and reproducibility of the body profile model for OSA patients.

**Table 3:** The cross validation and testing results of the body profile model on three groups—All patients, males only and females only. V: Cross Validation (TMUH); T: Testing (SHH); AHI: Apnea hypopnea index; Acc: Accuracy; Sen: Sensitivity; Spe: Specificity; AUC: area under curve; LR+: positive likelihood ratio; LR-: negative likelihood ratio; n: case number.

		Female		Male	
		*AHI ≥15 vs AHI <15*	*AHI ≥30 vs AHI <30*	*AHI ≥15 vs AHI <15*	*AHI ≥30 vs AHI <30*
V	Sen, %	**82.2±7.5**	**91.3±4.8**	**66.5±7.6**	**66.4±3.1**
	Spe, %	**76.1±4.8**	**76.6±2.9**	**87.4±16.6**	**81.7±3.7**
	Acc, %	**78.0±3.5**	**78.7±2.5**	**72.5±2.1**	**73.5±2.2**
	AUC, %	**87.8±3.4**	**91.8±1.7**	**84.9±1.9**	**81.0±1.8**
	LR+	**3.55±0.66**	**3.96±0.53**	**7.8±4.47**	**3.78±0.86**
	LR-	**0.23±0.1**	**0.11±0.06**	**0.37±0.08**	**0.41±0.04**
	N	**383 vs 859**	**178 vs 1064**	**2862 vs 1141**	**2132 vs 1871**
T	Sen, %	**74.8**	**78.3**	**62.5**	**68.3**
	Spe, %	**68.1**	**74.0**	**70.5**	**73.9**
	Acc, %	**70.1**	**74.6**	**65.4**	**71.4**
	AUC, %	**80.4**	**83.0**	**72.8**	**76.5**
	LR+	**2.34**	**3.01**	**2.12**	**2.62**
	LR-	**0.37**	**0.29**	**0.53**	**0.43**
	N	**103 vs 235**	**46 vs 292**	**518 vs 298**	**360 vs 456**

### The geriatricity-dependent model

Although age had been included in the body profile model, for the clinical needs and based on the physiological fact, we further established an geriatricity-dependent prediction model for the OSA severity. We divided the male group and female group into two subgroups, one of which was younger than 50 years old and the other older. The distribution of demographic information and collected parameters depending on age are listed in Table 4. The absolute value of correlation of BQ with the AHI was less than 0.1 in all subgroups. The ESS in the young male, older male, young female, and older female groups had the respective AHI correlations of 0.19, 0.31, 0.05, and 0.23. We thus established a body profile model for each subgroup based on the same four parameters: BMI, Neck, Waist, and Age.

**Table 4:** The demographic information and collected parameters in the TMUH and SHH databases depending on age. AHI: Apnea hypopnea index; BMI: Body mass index; BQ: Berlin questionnaire; ESS: Epworth sleepiness scale; #,*: the p-value is less than 0.05.

	TMUH				SHH			
	Male ≤ 50 y/o	Male > 50 y/o	Female ≤ 50 y/o	Female > 50 y/o	Male ≤ 50 y/o	Male > 50 y/o	Female ≤ 50 y/o	Female > 50 y/o
Number (total)	2616	1387	758	484	522	294	163	175
Number (AHI <15)	818	323	606	253	204	94	130	105
Number (30≥AHI>15)	445	285	84	121	85	73	19	38
Number (30≥AHI)	1353	779	68	110	233	127	14	32
AHI (events/hour)	37±30^#^	37.5±25.2^#^	9.8±18*	19.5±19.2*	32.2±28.5	32.5±25.6	10.5±16.1*	17.6±17.1*
Age (years)	37.7±7.5^#^	59.8±7^#^	36.1±8.3*	60±7*	38.7±7.3^#^	60.5±7.9^#^	37.9±8.7*	60.3±7.3*
ESS	10.1±5.5	10.2±6.2	8.5±5.4	8.7±5.9	10.4±5.3	10.4±5.9	9.9±5.5	9.2±6.5
Neck (cm)	39.2±3.6^#^	38.8±3.2^#^	33.3±3.6*	33.5±3.2*	38.8±3.6	38.6±3.4	33.6±3.6	33.4±3
Waist (cm)	94.4±13.2	93.7±10.3	81.2±16*	82.8±11.2*	95.9±13	94.6±10.9	85.1±12.8	86.2±11.1
BMI (kg/m^2^)	27.6±5.4^#^	26.3±3.8^#^	25.1±7.3*	24.8±4.3*	28.3±5.4^#^	26.5±4.1^#^	26±5.6	25.6±4.6

The cross validation results from the TMUH database and the testing results on the SHH database are shown in Table 5. The prediction accuracies of the young and older groups were significantly different in both male and female populations. The results in the testing group show that the prediction accuracy was higher in the young female subgroup, in particular when we distinguished the patients with severe or moderate OSA. The same finding holds for the male subgroups, with the accuracy being significantly higher in the young male subgroup. Moreover, the prediction accuracy of the female young group was significantly higher than that of the male young group. These testing results justify the applicability and reproducibility of the age-dependent body profile model to select patients with severe OSA.

**Table 5:** The cross validation and testing results of the age-dependent body profile model on four subgroups—Females younger than 50 years old, females older than 50 years old, males younger than 50 years old, and males older than 50 years old. V: Cross Validation (TMUH); T: Testing (SHH); AHI: Apnea hypopnea index; Acc: Accuracy; Sen: Sensitivity; Spe: Specificity; AUC: area under curve; LR+: positive likelihood ratio; LR-: negative likelihood ratio; n: case number.

		Female				Male			
		≤50 y/o		>50 y/o		≤50 y/o		>50 y/o	
		*AHI ≥15 vs AHI <15*	*AHI ≥30 vs AHI <30*	*AHI ≥15 vs AHI <15*	*AHI ≥30 vs AHI <30*	*AHI ≥15 vs AHI <15*	*AHI ≥30 vs AHI <30*	*AHI ≥15 vs AHI <15*	*AHI ≥30 vs AHI <30*
V	Sen, %	**82.7±7.9**	**95.3±9.0**	**76.2±10.0**	**90.9±9.5**	**72.0±10.8**	**75.1±8.2**	**63.7±5.3**	**63.5±10**
	Spe, %	**86.0±3.9**	**82.7±4.3**	**73.8±7.3**	**66.3±15.0**	**79.2±29.9**	**74.9±22.7**	**87.8±6.4**	**72.9±15.6**
	Acc, %	**85.3±3.5**	**83.7±4.1**	**74.9±5.9**	**71.9±11.2**	**74.3±2.6**	**75.0±7.3**	**69.2±4.4**	**67.6±4.8**
	AUC, %	**90.3±3.2**	**94.1±2.8**	**79.3±6.8**	**87.5±6.2**	**85.1±2.7**	**82.4±5.5**	**81.9±3.8**	**74.4±4.1**
	LR+	**6.4±2.1**	**5.96±1.94**	**3.14±1.03**	**3.02±0.61**	**7.03±1.34**	**4.02±0.64**	**6.59±3.24**	**2.69±1.04**
	LR-	**0.2±0.09**	**0.06±0.11**	**0.33±0.14**	**0.14±0.14**	**0.35±0.03**	**0.33±0.04**	**0.42±0.07**	**0.5±0.1**
	N	**152 vs 606**	**68 vs 690**	**231 vs 253**	**110 vs 374**	**1798 vs 818**	**1353 vs 1263**	**1064 vs 323**	**779 vs 608**
T	Sen, %	**75.8**	**92.9**	**72.9**	**71.9**	**68.2**	**73.4**	**61.0**	**66.9**
	Spe, %	**76.9**	**73.8**	**63.8**	**70.6**	**73.5**	**73.4**	**62.8**	**69.5**
	Acc, %	**76.7**	**75.5**	**67.4**	**70.9**	**70.3**	**73.4**	**61.6**	**68.4**
	AUC, %	**85.0**	**89.5**	**78.1**	**77.7**	**75.7**	**77.8**	**63.8**	**73.3**
	LR+	**3.28**	**3.55**	**2.01**	**2.45**	**2.58**	**2.75**	**1.64**	**2.19**
	LR-	**0.32**	**0.1**	**0.43**	**0.4**	**0.43**	**0.36**	**0.62**	**0.48**
	N	**33 vs 130**	**14 vs 149**	**70 vs 143**	**32 vs 143**	**318 vs 204**	**233 vs 289**	**200 vs 94**	**127 vs 167**

In conclusion, based on the above results, we could select the model based on both the gender and geriatricity for the clinical usage. Particularly, the body profile model works the best in the young female subgroup.

## Discussion

In this paper, we establish applicable predictors for OSA severity. The predictors were established based on the three easy-to-obtain anthropometric features such as waist circumference, neck circumference and BMI. The model was confirmed with high prediction rates on two independently large databases. It was found to be particularly accurate in discriminating patients with AHI higher or lower than 30 in the Asian young female group. Note that in this group, the negative likelihood ratio is consistently less than 0.1 both in the training and testing databases, which indicates an useful screening tool to rule out severe OSA in clinics. The result is slightly worse in discriminating patients with AHI higher or lower than 30 in the Asian young male group, but it also indicates that the model is a reasonable screening tool to rule out moderate and severe OSA. With the pre-test probability of OSA and the available likelihood ratio, the reported result allows us to determine th screening policy based on the likelihood ratio nomogram. However, as far as the authors know, a scientific report regarding the prevalence of OSA in Asia population is not available, except a literature summary paper [13]. In addition to the OSA prediction model, this paper also provides large-scale statistics for Asian body profiles compared with the existing reported statistics.[27]

The prediction model and its accuracy, however, still require discussion. First, we can see that the commonly applied questionnaires, including the BQ and ESS, do not correlate with OSA severity, and hence they are not included in the model. The low efficiency of the BQ is perhaps to be expected since it was designed based on data from Caucasian subjects while our databases consist of Asian subjects. This low efficiency once more suggests the importance of taking race into consideration when designing a predictor for OSA. The low efficiency of ESS, on the other hand, can be explained by the fact that these questionnaires were originally designed for subjective sleepiness rather than OSA screening. Since the information provided by BQ and ESS are not directly related to the body profiles, we expect to have higher prediction accuracy if there were Asian-based sleep apnea related questionnaires, and include them in the prediction model.

Second, it is clear that to distinguish patients with an AHI cutoff set at 15 or 30, the prediction model should be trained separately for males and females. While the model could be trained accurately for the female group, we could not train it to perform that well for the male group. It has been reported that, even with similar BMI and waist circumference, men with OSA have greater upper body (chest and abdomen) obesity,[28] which suggests that fat distribution may be important in the development of OSA. Other studies have directly observed more neck fat in male than female OSA patients.[29] These studies support our findings that anthropometric features reveal different amounts of OSA severity information in men and women.

Third, we also found that in both the male group and female group, the predictors perform better in the subgroup of patients younger than 50 years old. Note that as aging occurs, the upper airway structure, like connectivity tissue in general, undergoes changes such as the loss of collagen fibers or the downgrading of neuromuscular control of upper airway muscle tension.[30] Since anthropometric features might not faithfully reflect these upper airway changes, this might explain why the predictors perform worse in the older group. This difference is more significant in the female group, and this might be explained by the increasing proportion of android fat distribution[31] and the respiratory drive change caused by hormone control changes after menopause.[32]

Fourth, note that the testing step is carried out on an independent database, which is collected from a different hospital. Although following the same protocol, there are inevitable discrepancy and variation between two databases, which leads to a higher error rate in the testing database. The result indicates the robustness of the proposed prediction model, and hence its clinical potential.

While the accuracy of the proposed predictors is good, it could be further improved by combining other easy-to-obtain indicators. For example, as OSA is related to different diseases, the disease-related parameters that are already available from clinical information could be incorporated into the index for the final decision. This is a direction we will investigate in future work.

In addition to applying the developed model to screen OSA, it could be applied to the clinics to assist physicians. Based on the advanced computer vision tools, under proper patient privacy protection, we could develope a camera-based monitoring system to automatically estimate the body profile parameters in clinics. Such a system could be implemented on a mobile device (e.g., cell phone). We leave this potential research direction to the future work.

A discussion of this study is not complete without mentioning its limitations. First, only Asians are included in the databases. Since the body shape profiles of different races are known to be different,[33] corrections and hence other studies are needed to extend the proposed model to other races. Second, although we have carefully eliminated possible interfering factors by using techniques like cross validation, the fact that this study is a retrospective study could influence its results. Specifically, this is a stratified sample of the general population since the collected subjects were referred for the sleep testing due to some sleep problems. Also, note that while the buttock parameter has a good correlation with the outcome, since SHH does not contain the buttock parameter, we do not take it into account in the model. A well-designed prospective study is thus needed to confirm the proposed predictors for clinical usage. In the implementation, the optimization over different parameters for SVM, like the box constraint, the scaling factor of the kernel, and the threshold, could be considered to increase the accuracy of the final prediction model. We could also consider other supervised learning algorithms discussed in [24], or the recently proposed selective ensemble idea, like D3C [34], to further explore the structure inside the dataset. Third, in the testing SHH database, the number of older females (over 50 years old) is highly under-sampled compared with that of the other groups–hence the highly variable sensitivity and specificity values. This limitataion means that the finding of the predictor being better on the females younger than 50 years still needs to be confirmed with additional data. Last but not the least, we mention the potential important next step to establish prediction models for different races, like Caucasians, Africans, Indians, Hawaiians, etc. However, due to the lack of data, we could not conclude it in this study.

## Conclusion

This study shows that we could establish an applicable prediction model for the severity of obstructive sleep apnea in Asians by simply considering their anthropometric features and age, with better accuracy by dividing the patients’ population into subgroups based on different genders and age. It provides a new approach to extract the complicated relationship between the parameters influencing the severity of obstructive sleep apnea.

## Supporting information

S1 Dataset The database from TMUH sleep center. (XLSX)
